# Cefazolin/BMP-2-Loaded Mesoporous Silica Nanoparticles for the Repair of Open Fractures with Bone Defects

**DOI:** 10.1155/2022/8385456

**Published:** 2022-09-20

**Authors:** Mingkui Shen, Lulu Wang, Li Feng, Chuangye Xu, Yi Gao, Sijing Li, Yulan Wu, Guoxian Pei

**Affiliations:** School of Medicine, Southern University of Science and Technology, Shenzhen, 518055 Guangdong, China

## Abstract

The study aimed to explore the feasibility of a nanodrug delivery system to treat open fractures with bone defects. We developed a cefazolin (Cef)/bone morphogenetic protein 2 (BMP-2)@mesoporous silica nanoparticle (MSN) delivery system; meanwhile, Cef/MBP-2@ poly(lactic-co-glycolic acid) (PLGA) was also developed as control. For the purpose of determining the osteogenic and anti-inflammatory actions of the nanodelivery system, we cultured bone marrow mesenchymal stem cells (BMSCs) and constructed a bone defect mouse model to evaluate its clinical efficacy. After physicochemical property testing, we determined that MSN had good stability and did not easily accumulate or precipitate and it could effectively prolong the Cef's half-life by nearly eight times. In BMSCs, we found that compared with the PLGA delivery system, MSNs better penetrated into the bone tissue, thus effectively increasing BMSCs' proliferation and migration ability to facilitate bone defect repair. Furthermore, the MSN delivery system could improve BMSCs' mineralization indexes (alkaline phosphatase [ALP], osteocalcin [OCN], and collagen I [Col I]) to effectively improve its osteogenic ability. Moreover, the MSN delivery system could inhibit inflammation in bone defect mice, which was mainly reflected in its ability to reduce the release of IL-1*β* and IL-4 and increase IL-10 levels; it could also effectively reduce apoptosis of CD4^+^ and CD8^+^ T cells, thus improving their immune function. Furthermore, the percentage of new bones, bone mineral density, trabecular volume, and trabecular numbers in the fracture region were improved in mice treated with MSN, which allowed better repair of bone defects. Hence, Cef/BMP-2@MSN may be feasible for open fractures with bone defects.

## 1. Introduction

Recent years have witnessed a rising incidence of multiple and compound injuries that are mostly complicated with open limb fractures due to increasing traffic accidents [1]. As patients' fracture ends are combined with bone defects and are easily polluted, delayed healing and even chronic osteomyelitis may occur at the fracture end during the long-term healing process [2]. To achieve stable fixation effects and promote rapid recovery from the disease, it is often necessary to select appropriate materials to fill the bone defects. At present, mainly autologous and allogeneic bones are used for filling, with which good clinical results have been achieved [3]. However, there are also deficiencies. For example, autologous bone transplantation can cause injuries on the bone removal site [4], whereas transplantation of allogeneic bone may lead to rejection [5]. Therefore, the development of filling materials with bone induction and bone conduction ability would be key to treating open fractures complicated by bone defects. In addition, although these wounds have been thoroughly debrided, in large and deep wounds, a few foreign bodies might accumulate in both soft and bone tissue, resulting in wound inflammation and even osteomyelitis later in the healing process [6]. Therefore, if the filling material would have antibacterial effects or could include antibiotics, it could have an effective sterilizing effect in the marrow cavity and could effectively promote wound healing. Recent progress in tissue bioengineering has allowed solving the above issues.

In tissue bioengineering, seed cells, scaffold materials, and associated growth factors are often combined to repair bone defects [7]. Bone marrow mesenchymal stem cells (BMSCs), with potent repair ability and multidirectional differentiation potential, are commonly used as seed cells for bone repair [8]. Therefore, stimulating the effective BMSC differentiation into osteoblasts (OBs) is the key to promoting the repair of bone defects [9]. Bone morphogenetic protein 2 (BMP-2) has been reported to effectively shorten the healing time of fractured mice and increase bone mineral density (BMD) and trabecular thickness at the bone defect [10]. In a follow-up study, the BMP-2-activated SMDA signaling pathway was found to promote related functional protein levels, thus enabling BMSCs' differentiation into OBs, the initiation of the entire osteogenesis process [11, 12]. Therefore, the combination of BMP-2 and BMSCs can effectively enhance the proliferative and osteogenic ability of BMSCs, which could, in turn, promote bone defect healing [13, 14].

To effectively colonize BMP-2 in the marrow cavity and to achieve continuous release, we chose mesoporous silica nanoparticles (MSNs) as BMP-2's transmission carrier. MSN has a large spatial structure inside, which can effectively load BMP-2 [15]. Simultaneously, its adjustable pore structure can well regulate the release rate of BMP-2 to be continuous in the marrow cavity [16]. In addition, MSN has good modifiability [17]. By modifying a variety of superficial functional groups, MSN can achieve pH response characteristics and anti-inflammatory effects [18, 19] to further improve the transmission efficiency and curative effect of the loaded drugs.

In addition, chronic inflammation will stimulate osteoclasts to increase their activity, inhibiting the formation of a new bone [20]. Due to deep wound and serious tissue pollution, bacterial infection is easy to occur in the therapy of open fracture with bone defect; therefore, systemic antibiotic treatment is required at the same time. Cefazolin (Cef), as the first generation *β*-lactam antibiotics, can destroy the bacterial cell wall to kill them. Cef has good sensitivity to varieties of G^+^ bacteria and can also inhibit G^−^ bacterial infection, which is one of the representative antibiotics in clinic and has a wide range of clinical indications. Unfortunately, the systemic use of antibiotics can hardly reach an effective inhibitory concentration in the marrow cavity. A previous study reported Cef concentration in infected bone tissue approximately 10 times lower than that in serum [21]. In addition, bacteria will adhere to the implant surface to form a biofilm to further resist the penetration of Cef, thereby reducing the clinical therapeutic effect. To overcome the above limitations of Cef and minimize the risk of treatment failure, we loaded Cef into MSN, which can effectively reduce losses during transportation and can reduce the adverse reactions caused by excessive systemic use. Conversely, it is more conducive to reaching effective inhibitory concentration in the marrow cavity and effectively inhibits the inflammatory reaction, which could increase the treatment outcome of the MSN delivery system for open fractures complicated by bone defects [22]. In this study, we investigated the role of Cef and BMP-2-loaded MSN in repairing open fractures complicated by bone defects.

## 2. Data and Methods

### 2.1. Cef/BMP-2@MSN Preparation

#### 2.1.1. MSN Synthesis

After dissolving hexadecyltrimethylammonium bromide (CTAB) (3 g; H5882-100G, Sigma-Aldrich, USA) in 300 mL of dH_2_O, the pH of the solution was prepared to 11 by adding NaOH. Following 15 min of stirring that maintained at 50°C, tetraethyl orthosilicate (TEOS) (3 g; 333859-25ML, Sigma-Aldrich) was further placed to the above mixture for another 2 h of stirring. The supernatant was removed after centrifugation (3,000 rpm), and the resulting precipitate was treated with two rinses with dH_2_O and alcohol. Then, the CTAB in the above particles were removed by continuous thermal (550°C) degradation for 4 h. After vacuum drying, MSNs were collected and its FTIR spectrum was measured by ATR.

#### 2.1.2. Cef and BMP-2 Loading into MSNs

**Figure 1:**
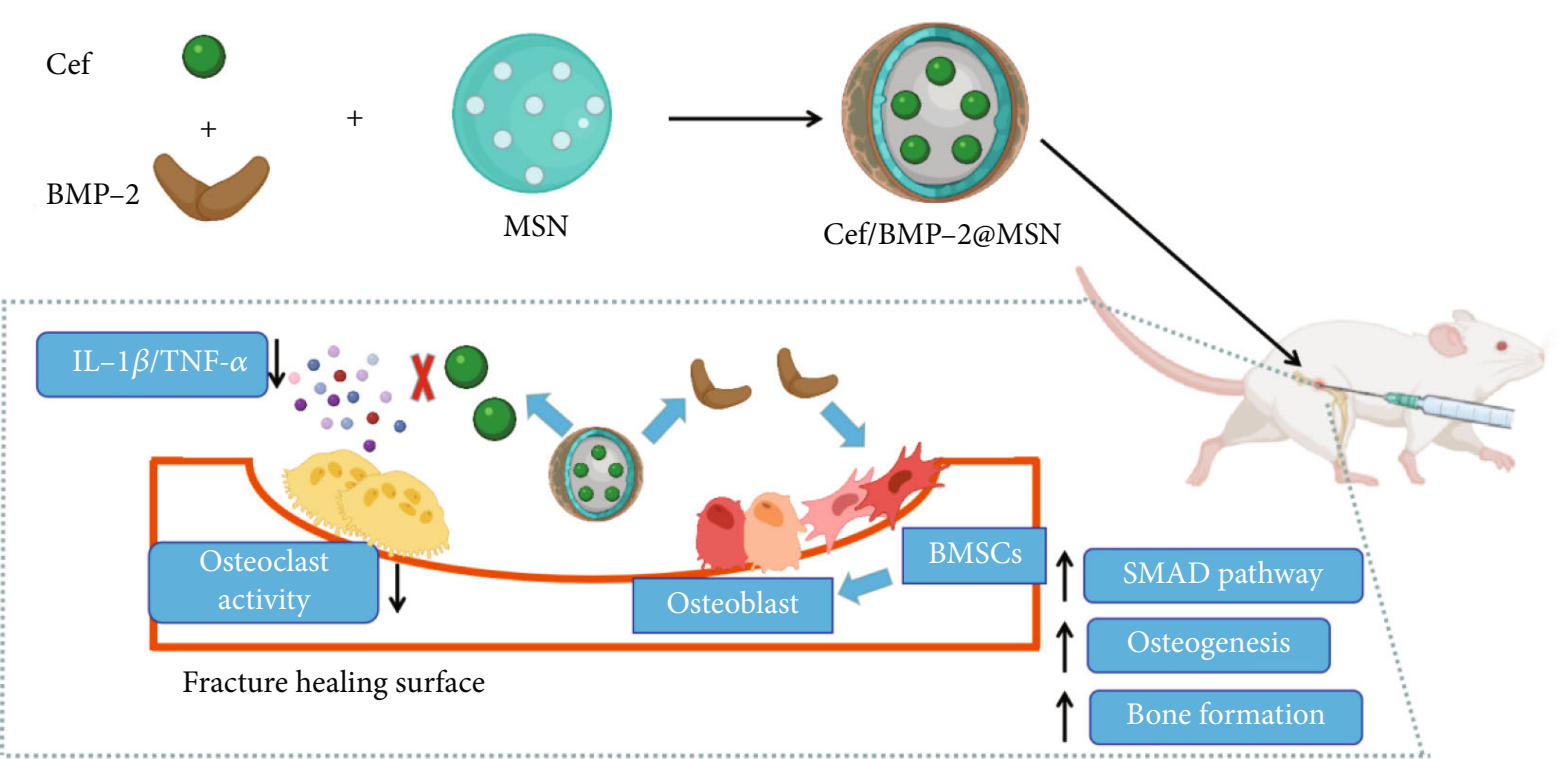
Schematic diagram of the assembly process and mechanism of Cef/BMP-2@MSN.

Cef (20 mg) was dissolved in acetate buffer at pH 5 and adjusted to 10 mg/mL. Then, 200 mg of MSN was dissolved in 4 mL of the above configuration solution. After magnetic stirring (300 rpm) for 2 h at 26°C, Cef was loaded into MSN using the concentrated solution adsorption method, and then, 1 mL of BMP-2 solution (10 mg/mL; ab80798, Abcam, USA) was put into the above mixture. After ultrasonic emulsification for 5 min, Cef/BMP-2@MSNs were obtained by vacuum drying, and they were washed twice with PBS (Figure 1). Cef/BMP-2@MSN was then freeze-dried and finally stored at 4°C.

Meanwhile, based on the conventional protocol, we assembled poly(lactic-co-glycolic acid) (PLGA) (S24436, Shanghai Yuanye Bio-Technology Co., Ltd) with Cef and BMP-2 to build the BMP-2@PLGA delivery system as a control. Briefly, 20 mg of Cef was dissolved in methanol solution (1:2) and added to PLGA liquid dissolved in dichloromethane (270997, Sigma-Aldrich). After magnetic stirring (35000 rpm, 40 s), the above polymer solution was added into PVA (0.5%, 3 mL, 38534, Sigma-Aldrich) for further emulsification, and then, 1 mL of BMP-2 solution (10 mg/mL) was placed to the above mixture. After further emulsification of the mixture for 5 minutes, dichloromethane was wiped off with the help of rotary evaporation (RV-211M, Shanghai Yiheng Scientific Instrument Co., Ltd., China) [23, 24]. At last, the above emulsion solution was vacuum dried to obtain Cef/BMP-2@PLGA, and they were washed three times with dH_2_O and freeze-dried at 4°C.

### 2.2. Cell Culture

Mice BMSCs (MUBMX-01001, Cyagen, Guangzhou, China) planted in the wells of 24-well plates were cultivated for 24 h with mice BMSC specific medium (MUXMX-90011, Cyagen) placed in a cell incubator (51032124, Thermo Fisher Scientific, Waltham, MA, USA) under 5% CO_2_ and at 37°C. The cultured cells were then diluted to a 4 × 10^4^/mL solution for further use.

### 2.3. Bone Defect Mouse Model Building

Nine 8-week-old male C56BL/6 J mice (Jiangsu Jicui Yaokang Biotech, China), weighting 120–150 g, were purchased for bone defect animal model building. After anesthesia with ketamine, a 2 cm incision was created on the mouse thigh skin and the femur was fully exposed through layer-by-layer cutting of the subcutaneous tissue. The middle femur was cut off with a wire saw, and the space between fracture ends was expanded. Then, a 25 g needle was inserted into the femoral marrow cavity for fixation, and the wound was sutured layer by layer. Postoperatively, the mice were kept warm on a heating pad until they could move again. Eventually, bone defect mice were successfully modeled. The above mouse models were further assigned to the control, PLGA, and MSN groups, for saline, Cef/BMP-2@PLGA, and Cef/BMP-2@MSN injection into the lesion site every three days for 2 weeks, respectively. The experiment, ratified by the Experimental Animal Ethics Committee of Medical College of South University of Science and Technology, was carried out following the Chinese guidelines for the care and use of laboratory animals.

### 2.4. Physicochemical Characterization of Cef/BMP-2@MSN

#### 2.4.1. Stability Test

The stability of an in vivo nanodelivery system is important for smooth drug delivery to the target. In this study, Cef/BMP-2@MSN was immersed in PBS and PBS + 10% serum to simulate the in vivo environment, and the size of the Cef/BMP-2@MSN particles was observed under a scanning electron microscope (SP8, Leica, Wetzlar, Germany). Further, to better show the in vivo stability of the MSN delivery system, Cef/BMP-2@MSNs were coupled with Cy5 and dissolved in PBS + 10% serum. Then, to test the production of sediment, their fluorescence intensity was observed at 0, 24, 48, and 72 h.

#### 2.4.2. Cef's Half-Life Determination

To determine the half-life of Cef, 20 mg of Cef/BMP-2@MSN and Cef/BMP-2@PLGA was mixed with PBS at 37°C and centrifuged (1,000 rpm) to collect the supernatant. Cef concentration was determined by HPLC.

#### 2.4.3. Permeability Test

Before testing Cef/BMP-2@MSNs' therapeutic effects, we tested their permeability in bone tissue by observing the fluorescence intensity of Cef/BMP-2@MSN-Cy5. Eight-week-old male C56BL/6J mice were divided into the MSN and PLGA groups that were subjected to Cef/BMP-2@MSN-Cy5 and Cef/BMP-2@PLGA-Cy5 injection into the bone marrow cavity of the mouse femoral shaft, respectively. After 24 h, the injection site was dissected for observation of Cy5-labeled cells under a confocal microscope (FV3000, OLYMPUS, Japan). Stronger infiltration capacity is indicated if the cells are more brightly labeled.

### 2.5. Cell Viability and Apoptosis Assays

The ability of BMSCs in repairing bone defects can be evaluated by detecting their proliferation activity and apoptosis. BMSCs, 1 mL of dilution as prepared in Section 2.2, were added to a 12-well plate with mouse BMSC specific medium and split into the control, PLGA, and MSN groups that were given no treatment, Cef/BMP-2@PLGA, and Cef/BMP-2@MSN, respectively. After culturing in a cell incubator for 24 h, we first observed the cell density under a differential interference contrast (DIC) microscope (MX8R, Dongguan Beitesen Precision Instrument Co., Ltd., China). Then, anti-ki67 and anti-caspase-3 (Abcam, USA; ab15580, ab32351) were used to label the active cells in each group and mark apoptotic BMSCs, respectively. Cell viability was observed with a confocal microscope.

### 2.6. Cell Migration Detection

To detect BMSC migration in the different groups, 1 mL of BMSC dilution from Section 2.2 was added to a 12-well plate with mouse BMSC specific medium and groups as the control, PLGA, and MSN groups for no treatment, Cef/BMP-2@PLGA intervention, and Cef/BMP-2@MSN intervention, respectively. After culturing in a cell incubator for 24 h, a pipette gun was used to scratch the cell layer, and then, the BMSCs on the scratch were washed out with PBS. After culturing with serum-free medium (10743011, Thermo Fisher Scientific, USA) for another 24 h, BMSC migration at the scratch was observed microscopically, and the total number of migrating BMSCs was calculated.

### 2.7. Mineralization and Osteogenesis Characterization

During bone repair, the mineralization and osteogenesis of BMSCs also play a vital part. Therefore, we measured their mineralization ability by immunofluorescence (IF) of alkaline phosphatase (ALP), osteocalcin (OCN), and collagen I (Col I). AP-TNAP ALPL polyclonal antibody Cy3 conjugated and OCN antibody FITC conjugated (both from Nanjing Chuanbo Biotech, China; C03321Cy3, C05448F) as well as anti-collagen I/FITC (Shanghai Weimeng Biotech, China) were used for staining on days 4 and 7 after culture, and the fluorescence intensity was observed using a confocal microscope. ALP, OCN, and Col I mRNA levels of each group were quantified by PCR. In brief, the total RNA of the above indicators in cells was isolated first, and then reverse transcribed into cDNA. *β*-Actin was chosen as the internal parameter of ALP, OCN, and Col I, and their relative expressions were calculated by 2^−△△Ct^ method. See Table 1 for the primer information.

### 2.8. Detection of Immunoinflammatory Responses in Our Mouse Model

To evaluate the inflammatory response in bone defect mouse models, tail venous blood of mice was extracted on the 4th day after treatment. The supernatant was collected via centrifugation (1,000 rpm) to detect IL-1*β*, IL-4, and IL-10 levels by ELISA. Mouse IL-1 beta ELISA Kit (ab197742), Mouse TNF alpha ELISA Kit (ab100747), and Mouse IL-10 ELISA Kit (ab255729) were purchased from Abcam. For immune function detection, the blood samples of mice were collected to separate peripheral blood mononuclear cells (PBMC) by density gradient centrifugation, and PBMC concentration was adjusted to 1 × 10^6^/mL with binding buffer solution. A PBMC suspension (100 *μ*L) was transferred to a 5 mL PE tube, and PE Anti-CD4 antibody [EPR20122] (ab252151) and Alexa Fluor® 647 anti-CD8 alpha antibody [EPR21769] (ab237365) were used to separately label CD4 and CD8. Then, 5 *μ*L of annexin V-FITC and 5 *μ*L of propidium iodide were placed to the above mixture solution. After mixing and incubating in the dark for 15 min, flow cytometry (Attune CytPix, Thermo Fisher Scientific) was used to detect lymphocyte apoptosis.

### 2.9. Therapeutic Effect Detection of Cef/BMP-2@MSN

Four and seven weeks after treatment, mice were killed and their femurs were excised and stored at −20°C. First, a macroscopic image of the specimen was observed, including callus formation and fracture line healing at the fracture end. Second, femoral specimens were scanned with microcomputed tomography (CT, Skyscan 1272, Bruker, Belgium), and the plane pixel resolution was set to 2,048 × 2,048, with a layer spacing of 16 *μ*m. The proximal fracture (a thickness of 0.5 mm) from scanned images was selected for detection, and the percentage of new bones, BMD, trabecular volume (TBV), and average trabecular number (TB.N) were observed. The BMD and percentage of new bones were obtained indirectly by voxel quantitation of CT scanning images.

### 2.10. Statistical Methods

SPSS 23.0 and prism were used for data processing. The inter-group differences of measurement data, denoted by ($\bar{x}\pm s$), were tested by the *t* test, with *P* < 0.05 indicating the presence of significance.

## 3. Results and Discussion

### 3.1. Characteristics of the Cef/BMP-2@MSN Delivery System

**Figure 2:**
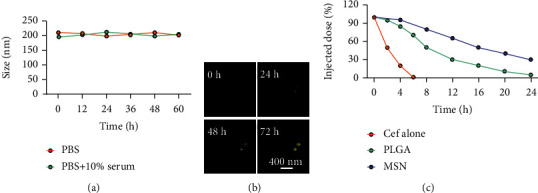
Physicochemical characterization of Cef/BMP-2@MSN. (a) Cef/BMP-2@MSN stability in different media. (b) Precipitate numbers of Cef/BMP-2@MSN-Cy5 standing in serum for certain hours. (c) Half-life period test of Cef in MSN and PLGA.

The FTIR spectrum of synthesized MSN showed that there were absorption peaks at 1095 cm^−1^ and 905 cm^−1^, which were anti-symmetric and symmetric stretching vibrations of Si-O-Si; meanwhile, the absorption peak at 968 cm^−1^ was the bending vibration of Si-OH, which was consistent with the FTIR spectrum of MSN previously reported [25]. The stability of the nanodelivery system can significantly improve drug delivery efficiency. As shown in Figure 2(a), the Cef/BMP-2@MSN was about 240 nm in size, with the ability to maintain a stable size in both the PBS and serum; only a few precipitates could be detected after fluorescein binding (Figure 2(b)). Otherwise, Cef's half-life significantly influenced the dosage and duration of drug administration. The nanodelivery system effectively prolonged Cef's half-life by eight times in the MSN group (Figure 2(c)). Thus, MSN can remain stable while improving the half-life of Cef during in vivo delivery.

### 3.2. Cef/BMP-2@MSN Could Penetrate the Bone Tissue

**Figure 3:**
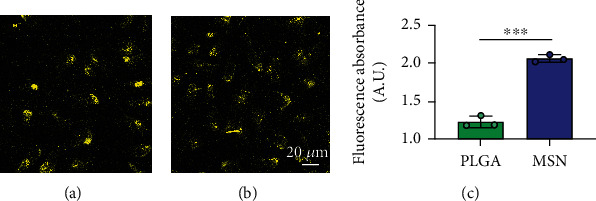
Infiltration of Cef/BMP-2@MSN in bone marrow tissue. (a) Infiltration of PLGA. (b) Infiltration of MSN. (c) Quantitative fluorescence evaluation of PLGA-Cy5 and MSN-Cy5 in bone tissue. ∗∗∗*P* < 0.001.

The permeability of nanodelivery systems in bone tissue is key to improve drug efficacy. As shown in Figures 3(a) and 3(b), more MSNs permeated the bone tissue than PLGA. Quantitatively, MSN showed higher fluorescence intensity than PLGS in bone tissue (Figure 3(c)). Therefore, MSN could better transfer Cef and BMP-2 to the bone tissue.

### 3.3. Cef/BMP-2@MSN Could Increase BMSC Activity

**Figure 4:**
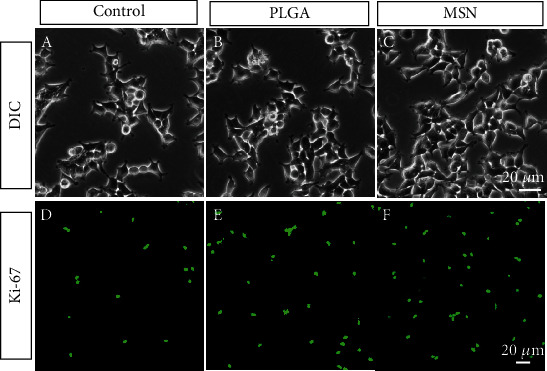
BMSC viability assessment. (a–c) DIC image of BMSCs in the control, PLGA, and MSN groups on day 7. (d–f) Anti-Ki67 expression pattern in the control, PLGA, and MSN groups on the 7th day.

A high BMSC activity can improve the efficiency of bone repair. As shown in Figures 4(a)–4(c), we found that BMSCs proliferated more and arranged more closely under DIC. Moreover, PLGA and MSN-treated BMSCs, especially MSN-treated ones, showed higher fluorescence intensity than the control group (Figures 4(d)–4(f)). The above results suggest a higher ability of MSN to promote BMSC proliferation.

### 3.4. Cef/BMP-2@MSN Could Reduce BMSC Apoptosis

**Figure 5:**
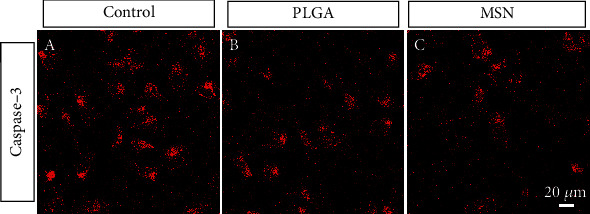
BMSC apoptosis detection. Expression patterns of anti-caspase-3 in the control group (a), PLGA group (b), and MSN group (c).

The apoptotic degree of BMSCs can reflect the repair ability of bone defects. The PLGA and MSN groups exhibited higher fluorescence intensities than the control group, especially the MSN group (Figure 5), demonstrating the ability of MSN to better suppress BMSC apoptosis.

### 3.5. Cef/BMP-2@MSN Could Increase BMSC Mobility

**Figure 6:**
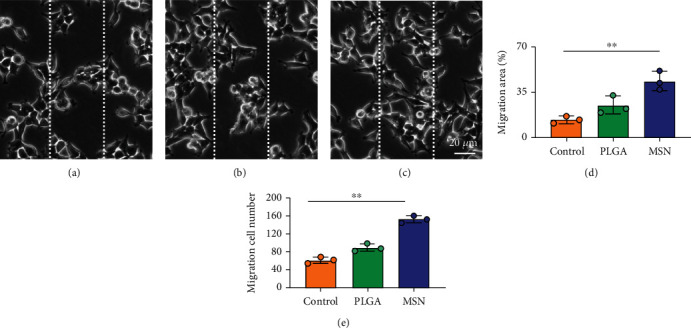
Scratch test of BMSCs in different groups. (a) BMSCs without any treatment. (b) BMSCs treated with PLGA. (c) BMSCs treated with MSN. (d) Quantitative evaluation of BMSC migration range in the three groups. (e) Quantitative evaluation of BMSC migration number in the three groups. ∗∗*P* < 0.01.

BMSC mobility can reflect the bone induction and bone conduction ability of nanodelivery systems. Compared with the control group (Figure 6(a)), more BMSCs migrated to the scratch area in the PLGA and MSN groups, especially in the MSN group (Figures 6(b) and 6(c)). Quantitative analysis showed that the MSN group had a higher number of migrating BMSCs and area of displacement than the control group (Figures 6(d) and 6(e)).

### 3.6. BMSC Mineralization and Osteogenesis Significantly Increased in the MSN Group

**Figure 7:**
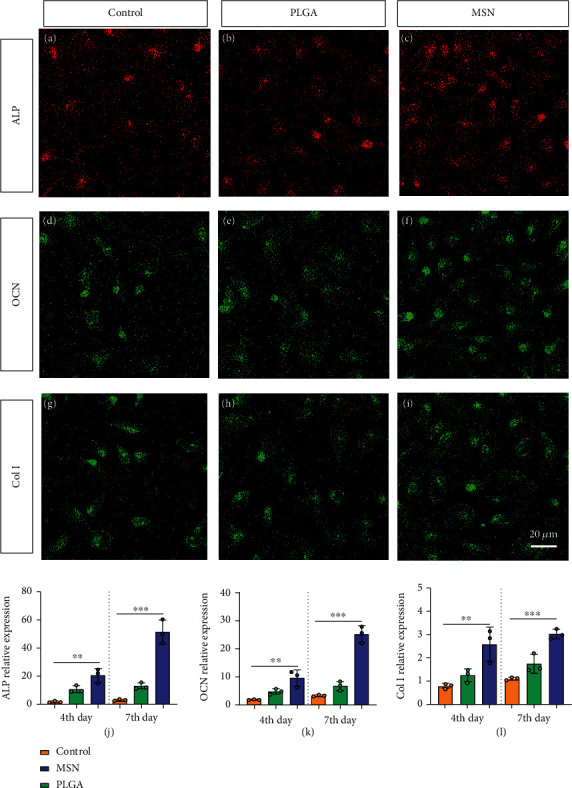
Osteogenic gene measurements. (a–c) ALP fluorescent staining. (d–f) OCN fluorescent staining. (g–i) Col I fluorescent staining. (j–l) Quantification of ALP, OCN, and Col I relative expression in the control, PLGA, and MSN groups. ∗∗*P* < 0.01; ∗∗∗*P* < 0.001.

BMSC mineralization and osteogenesis can effectively reflect the maturity of OBs. Hence, we evaluated the osteogenic capacity of the MSN delivery system by measuring the levels of osteogenic genes (ALP, OCN, and Col I). After 7 days of culture, the MSN group showed higher fluorescence intensities of those markers than the control and PLGA groups (Figures 7(a)–7(i)). Quantitative analysis also determined higher ALP, OCN, and Col I mRNA levels in the MSN group (Figures 7(j)–7(l)). It indicates that MSN can better increase the osteogenic capacity of BMSCs.

### 3.7. The Immune Function of Mice Significantly Improved in the MSN Group

**Figure 8:**
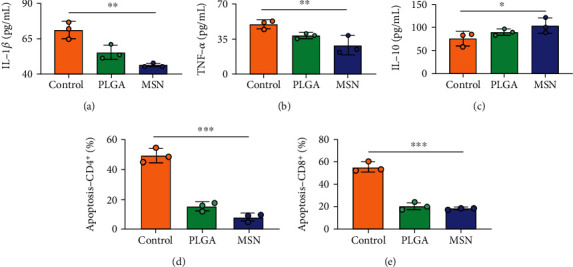
Immunoinflammatory response in mouse models. (a) Comparison of serum IL-1*β*. (b) Comparison of serum TNF-*α*. (c) Comparison of serum IL-10. (d) Comparison of CD4^+^ T cell apoptosis. (e) Comparison of serum CD8^+^ T cell apoptosis. ∗*P* < 0.05; ∗∗*P* < 0.01; ∗∗∗*P* < 0.001.

For the treatment of open fractures, inhibiting inflammatory reactions and improving immunity are important for promoting bone repair. IL-1*β* and TNF-*α* levels were lower, while IL-10 was higher in the MSN group compared with the control group (Figures 8(a)–8(c)). In terms of immune function, the levels of apoptotic CD4^+^ and CD8^+^ lymphocytes clearly decreased in the MSN and PLGA groups versus the control group (Figures 8(d) and 8(e)). Therefore, MSN is able to better transport Cef to bone tissue to inhibit inflammation and enhance immune function.

### 3.8. MSN Delivery System Could Better Accelerate Bone Defect Repair

**Figure 9:**
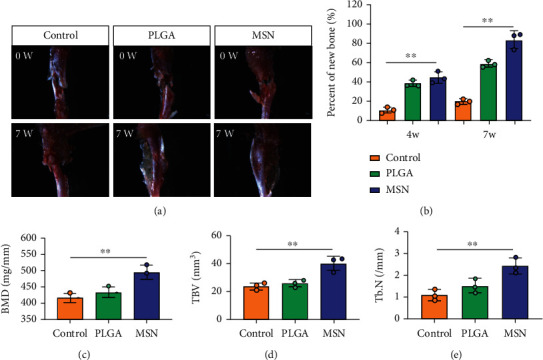
Therapeutic effects of Cef/BMP-2@MSN on bone defects. (a) Macroscopic findings of lesions in bone defect mice treated with saline, PLGA, or MSN. (b) Percentage of new bones at the fracture end in bone defect mice treated with saline, PLGA, or MSN. (c) BMD of lesions in bone defect mice treated with saline, PLGA, or MSN. (d) TBV of lesions in bone defect mice treated with saline, PLGA, or MSN. (e) TB.N of lesions in bone defect mice treated with saline, PLGA, or MSN. ∗∗*P* < 0.01.

We developed bone defect mouse models to further evaluate MSN's repair ability. As shown in Figure 9(a), MSN could better promote callus formation at the fracture end of bone defect mice and could blur the fracture line, and PLGA could only partly improve the lesions. Otherwise, we quantitatively evaluated the bone defect repair ability of MSN by the percentage of new bones, BMD, TBV, and TB.N, and found them improved in mice models treated with MSN (Figures 9(b)–9(e)). Therefore, MSN can better speed up bone defect repair.

## 4. Discussion

The development of bioengineering technology allows new therapeutic options for a variety of diseases, including open fractures with bone defects [26]. Biomaterials can be implanted into bone defects and can serve as a scaffold for osteoblast adhesion while improving bone induction and conduction [27]. Moreover, biomaterials can be loaded with antibiotics and growth factors according to the treatment needs to enhance the antibacterial and osteogenic effects of scaffold materials [28]. For example, Chatzipetros et al. [29] used composite scaffold material made of nanohydroxyapatite (nHA)/chitosan to fill and repair rat calvarial defects model. At 4 weeks after operation, a large number of new bones were found to form around the scaffold, which can promote the fusion of scaffold material and host bone and further ensure its stability. At 8 weeks after operation, massive new bone formed in the scaffold material, which further confirmed its bone induction and osteogenesis. In vitro test, Kuang et al. [30] further loaded levofloxacin on the three-dimensional composite scaffold made of MSN/nHA/polyurethane, which effectively improved the anti-infection ability of the scaffold and reduced the adhesion of bacteria, so as to give better play to its effect in bone defect repair. Therefore, biomaterials provide a new direction for treating open fractures complicated by bone defects.

In this study, we used MSN loaded BMP-2 and Cef to form composite scaffolds for bone defect treatment, and Cef/BMP-2@MSN was found to have good stability in serum and did not easily deposit, which could improve the delivery efficiency of Cef and BMP-2 in vivo. Therefore, Cef/BMP-2@MSN could better achieve the action target, which could better promote the differentiation of BMSCs and could inhibit the occurrence of an inflammatory reaction. In addition, the use of MSN increased the half-life of Cef by nearly eight times, which could effectively reduce the Cef dosage while ensuring its anti-inflammatory effects, while effectively reducing adverse reactions by drug overdose. Furthermore, MSN could better penetrate bone tissue, allowing BMP-2 to better promote BMSC differentiation into mature OBs. At the same time, it helped in reaching an effective inhibitory concentration of Cef in the bone tissue to better avoid the occurrence of osteomyelitis.

The good proliferative activity of BMSCs is conducive to better repair bone defects. In the MSN group, we found that BMSCs were more proliferative and closely arranged, and had stronger migration ability, which consists with the research results of Liu et al. [10]. The reason may be that compared with PLGA, MSN can better load BMP-2 and improve its targeted delivery function, so that BMP-2 can better penetrate bone tissue and exert its bone induction and bone conduction ability [31–33]. As a result, BMSCs in MSN group can be promoted to better differentiate into mature OBs, which is conducive to accelerating bone defect repair time and improving curative effects [34, 35]. To better quantify the degree of osteoblast differentiation and maturation, we evaluated the mineralization ability of BMSCs in terms of ALP, OCN, and Col I, and the levels of the above indicators increased significantly in the MSN group, indicating that the MSN delivery system could better play the osteogenic function of BMP-2 to accelerate bone repair.

In addition, the inflammatory response in mouse models treated with MSN was more effectively controlled, as shown by significantly lower IL-1 and IL-4 releases and a higher IL-10 release. Further, symptoms of congestion and edema at the fracture site of mice treated with MSN were better controlled. Szewczyk et al. [36] found that in osteomyelitis therapy, Cef@MSN drug delivery system had a better antibacterial effect on *Staphylococcus aureus* strains, which is consistent with our results. This is mainly due to the fact that MSN can better target and deliver the loaded Cef to bone tissue, so as to effectively increase the concentration of antibiotics around the scaffold to reduce the incidence of infection caused by open fracture. Moreover, the control of inflammation is helpful to reduce osteoclast stimulation and promoting bone defect repair. After CT scanning, the percentage of new bones at the fracture end in the MSN group was higher, so as BMD, TBV, and mean TB.N, which further confirmed that Cef/BMP-2@MSN had a significant effect on the repair of open fractures with bone defects.

This research was limited in that it only studied the proliferative activity and mineralization ability of BMSCs after BMP-2 stimulation but did not deeply explore its underlying mechanisms. In addition, the effects of different scaffolds on the repair of bone defects were not discussed. Therefore, the above deficiencies need to be further improved in follow-up research.

## 5. Conclusion

With favorable in vivo stability, the Cef/BMP-2@MSN delivery system can validly prolong the half-life of Cef. In addition, it can better penetrate bone tissue. Therefore, MSN can effectively reduce the dosage of Cef while ensuring good anti-inflammatory effects and improving the activity, migration ability, and mobility of BMSCs. In bone defect, the mouse that models the MSN delivery system alleviates inflammation and enhances immunity while promoting bone repair. Therefore, Cef/BMP-2@MSN may be a good choice for treating open fractures complicated by bone defects.

## Figures and Tables

**Table 1:** Primer information of PCR.

	F primer	R primer
ALP	5′-AACCCAGACACAAGCATTCC-3′	5′-CCAGCAAGAAGAAGCCTTTG-3′
OCN	5′-CAGGGCAGTAACTTATCTTG-3′	5′-CCTGAACCAAGCCTTACTCA-3′
Col I	5′-CACCCTCAAGAGCCTGAGTC-3′	5′-CGGGCTGATGTACCAGTTCT-3′
*β*-Actin	5′-CTGGCACCACACCTTCTACA-3′	5′-GGTACGACCAGAGGCATACA-3′

## Data Availability

The labeled dataset used to support the findings of this study are available from the corresponding author upon request.
